# Public Awareness of Medical Research Terminology in Japan, and the Accuracy of Physicians’ Predictions regarding that Awareness

**DOI:** 10.1007/s41649-023-00247-4

**Published:** 2023-03-30

**Authors:** Ayako Kamisato, Hyunsoo Hong, Suguru Okubo

**Affiliations:** 1 The Institute of Medical Science, The University of Tokyo, Tokyo, Japan; 2 Ritsumeikan University, Kyoto, Japan; 3 BMS Yokohama Inc., Yokohama, Kanagawa Japan

**Keywords:** Informed consent, Medical research terminology, Research ethics, Public awareness, Physicians’ predictions, Japan

## Abstract

One of the ethical principles of medical research involving human subjects is obtaining proper informed consent (IC). However, if the participants’ actual awareness of medical research terminology is lower than the researchers’ prediction of that awareness, it may cause difficulty obtaining proper IC. Therefore, this study aims to clarify the presence of “perception gaps” and then discuss IC-related issues and measures based on the insights obtained. We conducted two online surveys: a “public survey” to understand the Japanese public’s awareness of 11 medical research terms and a “physicians’ survey” to investigate physicians’ predictions regarding public awareness. In the “public survey,” for each term, respondents were instructed to select their situation from “understand,” “have heard,” or “have never heard.” In the “physicians’ survey,” respondents were asked to estimate the proportions of the general public who would “have understood,” “have heard,” or “have never heard” by using an 11-step scale. We analyzed separately in two age groups to understand the age-related difference. We received 1002 valid responses for the “public survey” and 275 for the “physicians’ survey.” Of the public respondents, more than 80% had never heard of terms such as *interventional study*, *prospective clinical study*, *cohort study*, *Phase I clinical trial*, or *double-blind study*. Concurrently, physicians overestimated general public awareness of the terms *placebo*, *cohort study*, *double-blind study*, and *randomized clinical trial* (in the *group of people under 60*). The results revealed the perception gap between the general public and physicians which raise serious concerns about obtaining proper IC from clinical research participants.

**Supplementary Information:**

The online version contains supplementary material available at 10.1007/s41649-023-00247-4.

## Introduction

Medical research involving human subjects is critical for the advancement of healthcare. However, the stipulated purpose of medical research, “to test a hypothesis, permit conclusions to be drawn, and thereby to develop or contribute to generalizable knowledge,” (National Commission for the Protection of Human Subjects of Biomedical and Behavioral Research 1979) makes it fundamentally different from medical services, which are intended to be beneficial for patients on an individual basis. Thus, medical research involving human subjects can only be conducted if ethical principles are met, such as obtaining proper informed consent (IC) (World Medical Association 2013; Emanuel et al. 2000). Proper IC is defined by three requirements: (i) researchers must provide complete information to research participant candidates (hereinafter called participants) for them to decide whether to participate in the study (*information*), (ii) participants must have an in-depth understanding of the provided information (*comprehension*), and (iii) participants must be free to decide whether to participate (*voluntariness*) (Dankar et al. 2019) (while there are opinions that these requirements should be reviewed in the first place (Millum and Bromwich 2021; Resnik 2021), however, we will not discuss those arguments here).

Each of these requirements, however, has some diverse issues. The primary issue with requirement (ii) is that many participants provide their consent without an in-depth understanding of the research. For example, a survey conducted by Tadros et al. indicated that among targeted individuals who participated in phase I clinical trials, only 7% were able to correctly answer the aims of the study, and only about 45% were aware of the primary side effects associated with the investigational drug (Tadros et al. 2019). A survey of patients who participated in a cancer-related clinical trial conducted by Schumacher et al. showed that even after receiving explanations related to the trial, over 80% of participants mistakenly believed that there were no differences in risks between the treatment they were given in the trial and the standard treatments (Schumacher et al. 2017). Further, a survey by Chu et al. that targeted patients who had participated in a clinical study conducted in Korea found that more than half of the respondents mistakenly believed that all participants in a clinical study could receive new drugs or treatments (Chu et al. 2012). These findings demonstrate the prevalence of serious ethical concerns. To address this situation, research that seeks to innovate explanatory methods is underway, including efforts to make IC forms more concise (Koonrungsesomboon et al. 2019), adding supplementary documents to IC forms or providing videos to promote participants’ understanding of the research (Kass et al. 2015; Palmeirim et al. 2020; Pal et al. 2021; Palmeirim et al. 2021; Synnot et al. 2014). As medical research becomes increasingly complicated (Getz and Campo 2017), such efforts are critically important. However, is the general public—many of whom may become potential research participants in the future—aware of basic terminology related to medical research, such as “interventional study” or “randomized clinical trial”? If the familiarity with these terms has not spread to the general public, it will be difficult for the average participant to fully understand the study, no matter how much the explanatory method for each study is devised. To find the answer to this question, we conducted a public survey in 2018, revealing public awareness and understanding of medical research terminology were generally low (Kamisato and Yoshida 2020). This result shows the necessity to know how physicians, who might explain medical research, predict the general public’s awareness of medical research terminology and whether there are some perception gaps between the general public and physicians. Since physicians have room to obtain proper IC if they can ascertain participants’ awareness levels and supplement their knowledge when explaining a study. In contrast, if physicians cannot ascertain participants’ actual levels of awareness, they cannot adapt their explanations accordingly. As a side note, in Japan, the support infrastructure for clinical research is relatively weak (Mukai 2013), and the number of clinical research coordinators and research nurses is inadequate (Ministry of Health, Labour and Welfare, Health Science Council 2019). Thus, in most cases at present, patients’ attending physicians are involved directly in clinical research efforts and provide necessary explanations to their patients about such research (Ito-Ihara et al. 2013). As a result, at least in Japan, it is critically important to understand physicians’ predictions. From such concerns, it is essential to investigate physicians’ predictions regarding public awareness of medical research terminology and to verify the presence of “perception gaps” between physicians and the general public. In particular, it is considered beneficial to divide the general public by age group—the elderly or young people—and analyze their characteristics. This is because the physician can know the participants’ approximate age at the time of explanation, and if there are characteristics in the awareness of medical research terminology according to the age group, physicians should change the content and method of explanation accordingly. However, very few previous studies in Japan or internationally have investigated the perception gaps between the general public and physicians, focusing on terms related to medical research (Yoshida et al. 2013). Therefore, this study aims to clarify the presence of “perception gaps” by focusing on age groups through online surveys, and also to discuss IC-related issues and measures based on the insights obtained.

## Materials and Methods

The data from two online surveys were used: a survey of the general public in 2018 (Public survey) and a survey of medical physicians in 2020 (Physicians’ survey). As mentioned above, we used the data from the “Public survey” to clarify the general public’s awareness level of medical research terminology and understanding level of it by using true or false questions (Kamisato and Yoshida 2020). However, in line with the physician survey that predicted awareness by age group of research participants, in this study, we divided respondents by age groups and then re-analyzed with a focus on the awareness level.

### Public Survey

#### Survey Method, Time Period, and Respondents

This survey was conducted online from 5 to 7 December 2018, by Intage Inc., a major online survey *company* in Japan (https://www.intage.co.jp/english/). The target number of responses was set to 1000. After considering the Japanese population composition, 7847 Japanese men and women between their 20s–70s, randomly selected from the company’s pool of registered survey-takers, were requested to participate. As we intended to discern conditions of perceptions among the average general public, we defined the “general public” as those who were not likely to touch with medical research terminology for occupational reasons. For this reason, individuals who worked or had family members working in the pharmaceuticals and health foods, medical care and welfare, mass media, advertising, or newspaper/broadcasting industries were excluded from the candidate pool. There were no special rewards from us, but respondents were awarded “points” that could be used for online shopping according to the company rules after completing the questionnaire.

#### Terminology

No previous studies have extensively investigated the awareness of medical research terminology among the general public in Japan. However, although the number of terms covered by each study was small, several previous studies have investigated on terms such as *clinical trial*, *chiken*, *placebo*, *informed consent*, *phase 1–3 trial*, and *double-blind trial* (Yoshida et al. 2013; Tsutani et al. 2015; Torigoe and Nakano 2013; National Institute for Japanese Language and Linguistics 2009). Thus, we selected 12 basic terms with reference to these terms and other terms from glossaries for patients (National Institute of Public Health n.d.; DIPEx Japan 2016). One of the terms, *chiken*, referring specifically to clinical trials performed to gain approval from regulatory authorities to manufacture/sell pharmaceuticals or medical devices, is a unique system in Japan. For this reason, in this article, we report results for the 11 terms listed below.

1. Clinical study
2. Epidemiological study
3. Interventional study
4. Prospective clinical study
5. Cohort study
6. Phase 1 clinical trial
7. Informed consent
8. Ethical review board
9. Double-blind study
10. Placebo
11. Randomized clinical trial

#### Survey Items

Only information regarding gender and age was used to grasp a holistic view of awareness concerning medical research terminology across the broader Japanese public.

According to a previous study that surveyed whether words such as “complications” and “antibodies” commonly used in a medical setting by physicians are understood by patients, one of the reasons for patients’ lack of understanding was their lack of prior awareness of these words (National Institute for Japanese Language and Linguistics 2009). Thus, in this study, we aimed to know the extent of awareness about the selected terms. For each term, respondents were instructed to select one of the following three statements: “I understand the meaning of this term,” “I have heard of this term before,” or “I have not heard of this term before.” The responses were categorized as *understand*, *heard*, and *never heard*, respectively. As it was unclear whether respondents who selected “I understand the meaning of this term” actually understood it correctly, we decided to avoid undue focus on this response. Then, we defined the two responses, “*understand*” and “*heard*,” as “aware” or “familiar,” meaning that the respondents recognized the term.

#### Analysis Method

Respondents were separated into two age groups for analysis to understand the age-related difference: working-age adults in their 20s–50s (*the group of people under 60*) and older adults in their 60s–70s (*the group of people 60s–70s*). Public awareness was gauged regarding the percentage of respondents endorsing each response and was separately calculated for each term and age group. Fisher’s exact test was employed to compare categorical variables in the statistical analysis, and probability values less than 5% (two-tailed) were deemed statistically significant. Power analysis was performed using G Power 3.1.

### Physicians’ Survey

#### Survey Method, Time Period, and Respondents

This survey was administered online from 13 to 16 March 2020, by the same online survey company that conducted the public survey. Of the Japanese physicians registered in this company, 1300 were requested to participate under the same conditions as the general public survey; that is, “points” were awarded if they completed the questionnaire according to the company rules.

#### Terminology

Given the study’s aim of determining perception gaps between the public’s awareness of medical research terminology and physicians’ predictions, the same 12 terms were selected. However, we omitted the term *chiken* in this article for the same reasons as in the public survey.

#### Survey Items

In addition to age and gender, respondents were asked about the type of medical institution they were working in (or, in the case of multiple workplaces, the one at which they had worked the longest), their department or specialty, and whether they had experience in medical research involving human subjects.

The respondents were requested to estimate how well each of the terms would be recognized by the general public. Initially, we asked respondents to make separate estimates for the two age groups—*the group of people under 60* and *the group of people 60s–70s* (hereinafter assumed age groups)—to gauge how physicians’ opinions differed depending on the participants’ ages. Then, physicians were asked for each term to estimate the proportions of each age group whom they believed would have likely (1) heard of the term before and understood its meaning (*likely understood*), (2) heard of the term before but not understood its meaning (*likely heard*), and (3) never heard of the term before (*likely never heard*). The nearest percentage on an 11-step scale (0%, 10%, …, 100%) was selected to provide the estimate. For example, the general public in *the group of people under 60*, those who fall under (1) are about 20%, those who fall under (2) are about 50%, and those who fall under (3) are 30%. Respondents were asked to choose their responses such that the three selected percentages summed up to 100%, and this requirement was stated in the questionnaire as a precaution. Responses that fulfilled this core prerequisite were considered valid. We could have used another methodology, including questions like “Do you think the general public would recognize the term *placebo*?” and instructed the respondents to select the response on a 5-point rating scale (5 = *I do not believe they would*, 3 = *neutral*, 1 = *I believe they would*) (Yoshida et al. 2013). However, it is not appropriate to bracket diverse people as “general public,” and it is easy to answer based on the specific “patient” or “citizen” that comes to mind of the respondents; therefore, the above answer method is adopted in this survey. The proportions estimated for *likely understood* and *likely heard* were combined into a single category (*expected awareness or familiarity*), corresponding to the category defined for the public survey.

#### Analysis Method

For the expected awareness of each terminology, we calculate the average value of the response rate (i.e., 0%, 10%, …, 100%) of each assumed age group. These values were subjected to statistical testing to compare the outcomes of *the group of people under 60* and *the group of people 60s–70s*. The Wilcoxon signed-rank test was utilized for statistical comparisons because the response data for expected awareness of the two age groups were provided by the same individual (i.e., physician). Endorsement rates of past contributions to clinical research among responding physicians were cross-tabulated and compared using Student’s *t*-test and Mann–Whitney *U* test. Furthermore, probability values of less than 5% (two-tailed) were considered statistically significant in all comparisons. Power analysis was performed using G Power 3.1.

### Ethical Issues

In both the public and physicians’ surveys, we displayed the summary in the browser window and asked the candidates to click the “start” button if they agreed to participate in the survey. In this way, we obtained consent to participate from all respondents. In addition, we did not receive participants’ personal information from Intage Inc. to safeguard the respondents’ privacy.

Our survey falls outside the scope of the Japanese government’s Ethical Guidelines for Life Science and Medical Research Involving Human Subjects, and there are no national guidelines in Japan for social and behavioral research. Therefore, this survey was conducted following the Ethical Principles for Sociological Research of the Japan Sociological Society.

## Results

### Public Survey

#### Demographic Characteristics

In total, 1002 valid responses were received out of 7847 survey requests (effective response rate: 12.8%). Respondents have a median age of 51 years (min: 20 years, max: 79 years). When divided according to age, 654 respondents were in *the group of people under 60* (65.3%), while 348 respondents were in *the group of people 60s–70s* (34.7%). Gender and age ratios are equivalent to those of the Japanese population at large (Ministry of Internal Affairs and Communications 2019). The respondents’ demographic characteristics and the Japanese population are presented in Table 1 for comparison.

**Table 1:** Public survey: Respondents’ attributes (*n* = 1002)

		Results of the survey	Japanese population as of 1 October 2018^a^
Gender	Male	493 (49.2%)	46,652,000 (49.6%)
	Female	509 (50.8%)	47,429,000 (50.4%)
Age (years)	20–29	120 (12.0%)	12,553,000 (13.3%)
	30–39	157 (15.7%)	14,630,000 (15.6%)
	40–49	200 (20.0%)	18,759,000 (19.9%)
	50–59	177 (17.7%)	16,011,000 (17.0%)
	60–69	187 (18.7%)	16,959,000 (18.0%)
	70–79	161 (16.1%)	15,166,000 (16.1%)

^*^Sums exceeded 100% in some cases because percentage data were rounded to the first decimal place

^a^Number and ratio to the target age (20s–70s) of this survey

#### General Public’s Awareness of Each Term

**Fig. 1:**
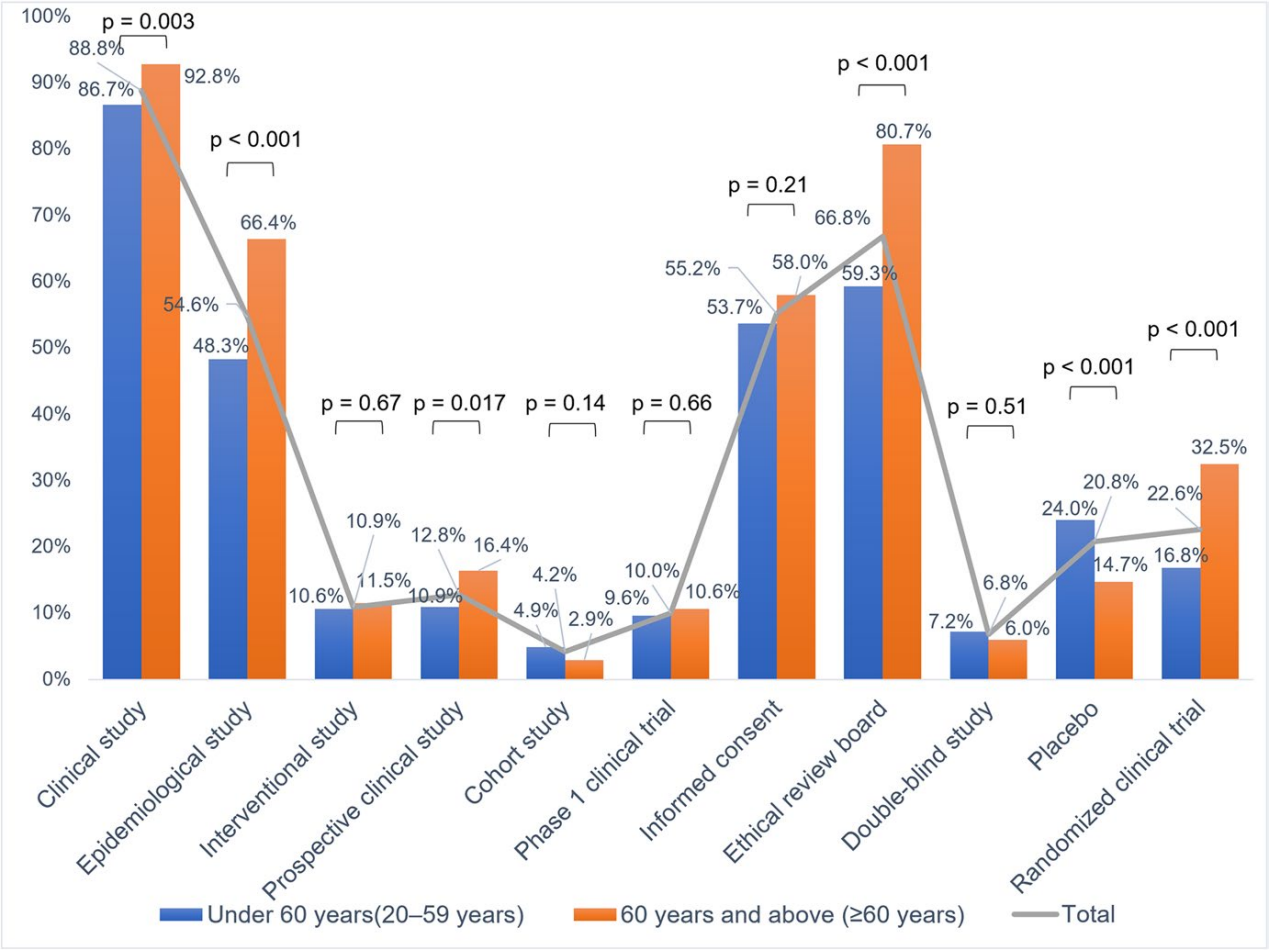
Public survey: Public awareness (“*understanding*” plus “*heard*”) by age group (*N* = 1002)

The term, *clinical study*, was the most familiar term among the 11 terms; around 90% of both age groups were aware of it. However, the second-most familiar term, *ethical review board*, was familiar to about 80% of respondents in *the group of people 60s–70s*, but only less than 60% of respondents in *the group of people under 60*. There were eight terms with less than 50% awareness among the public in *the group of people under 60* and seven terms among the public in *the group of people 60s–70s*. Among them, the terms whose awareness was less than 20% were *interventional study*, *prospective clinical study*, *cohort study*, *Phase 1 clinical trial*, *double-blind study*, and *randomized clinical trial* in *the group of people under 60*, and also *interventional study*, *prospective clinical study*, *cohort study*, *Phase 1 clinical trial*, *double-blind study*, and *placebo* in *the group of people 60s-70s*. This means that more than 80% of the general public has never heard of these terms. Comparing each term between two age groups, the following five terms—*clinical study*, *epidemiological study*, *prospective clinical study*, *ethical review board*, and *randomized clinical trial*—demonstrated statistical significance (*p*<0.05) for more familiarity among respondents in *the group of people 60s–70s*. In contrast, the only term that showed statistical significance (*p*<0.05) for more familiar respondents in *the group of people under 60* was “*placebo*” (Fig 1. Details are presented in Supplementary Table 1).

### Physicians’ Survey

#### Demographic Characteristics

From the 1300 requested surveys, we received 474 responses (36.5% response rate). Of them, 275 fulfilled the criteria for valid responses, such that the sum of the three percentages estimated for *likely understood*, *likely heard*, and *likely never heard* equaled 100% (effective response rate: 21.2%). Table 2 summarizes the demographic characteristics of these participants. Respondents have a median age of 54 years (min: 33, max: 70). Of the respondents, 82.5% and 17.5% are male and female, respectively. The department/specialty that respondents most commonly belonged to is “internal medicine,” and hospitals and clinics are the primary places of work for 63.7% and 30.2% of respondents, respectively. Comparing with the Ministry of Health, Labour and Welfare (2018) data regarding physicians in Japan, no noticeable difference, other than age distribution, was observed between the actual situation in Japan and these results regarding the sex ratio of physicians and the place of employment. In Japan, medical facilities having 20 or more beds are defined as “hospitals,” while those having 19 or fewer (or none at all) are defined as “clinics.” However, as seen in the small percentage of respondents in their 20s and 30s, the age distribution of the respondents in this survey was different from that of actual Japanese physicians. Furthermore, 60.4% of the respondents reported having previously contributed to medical research involving human subjects. Lastly, 27.1% of them worked at clinics, 65.7% at hospitals, and 7.2% at other healthcare facilities.

**Table 2:** Physicians’ survey: Respondents’ attributes (*n* = 275)

		Results of the survey	Statistics of Japanese physicians in 2018
Gender	Male	227 (82.5%)	255,452 (78.1%)
	Female	48 (17.5%)	71,758 (21.9%)
Age (years)	20–29	0 (0%)	29,605 (9.0%)
	30–39	17 (6.2%)	66,4955 (20.3%)
	40–49	70 (25.5%)	69,8625 (21.4%)
	50–59	117 (42.5%)	70,6105 (21.6%)
	60–69	69 (25.1%)	56,1945 (17.2%)
	≥ 70	2 (0.7%)	34,4445 (10.5%)
Medical institution (of employment)	Clinic	83 (30.2%)	103,836 (31.7%)
	Hospital	175 63.7%)	208,127 (63.6%)
	Other	17 (6.2%)	15,247 (4.7%)
Department/specialty	Internal medicine	93 (33.8%)	109,707 (35.2%)
	Surgery	29 (10.5%)	34,524 (11.1%)
	Orthopedic surgery	18 (6.5%)	21,883 (7.0%)
	Pediatrics	25 (9.1%)	18,158 (5.8%)
	Psychiatry/psychosomatic medicine	10 (3.6%)	16,842 (5.4%)
	Ophthalmology	10 (3.6%)	13,328 (4.3%)
	Obstetrics/gynecology	11 (4.0%)	13,276 (4.3%)
	Otorhinolaryngology	11 (4.0%)	9288 (3.0%)
	Rehabilitation medicine	1 (0.4%)	2705 (0.9%)
	Radiology	9 (3.3%)	6813 (2.2%)
	Dermatology	6 (2.2%)	9362 (3.0%)
	Pathology	1 (0.4%)	1993 (0.6%)
	Emergency medicine	4 (1.5%)	3590 (1.2%)
	Anesthesiology	11 (4.0%)	9661 (3.1%)
	Clinical testing	0 (0.0%)	604 (0.2%)
	Plastic surgery/cosmetic surgery	3 (1.1%)	3431 (1.1%)
	Neurology	9 (3.3%)	5166 (1.7%)
	Urology	7 (2.5%)	7422 (2.4%)
	Other	17 (6.2%)	24,210 (7.9%)
Past involvement in medical research on human subjects	Yes	166 (60.4%)	
	No	109 (39.6%)	

^*^Sums exceeded 100% in some cases because percentage data were rounded to the first decimal place

#### Physicians’ Prediction of Public Awareness

**Fig. 2:**
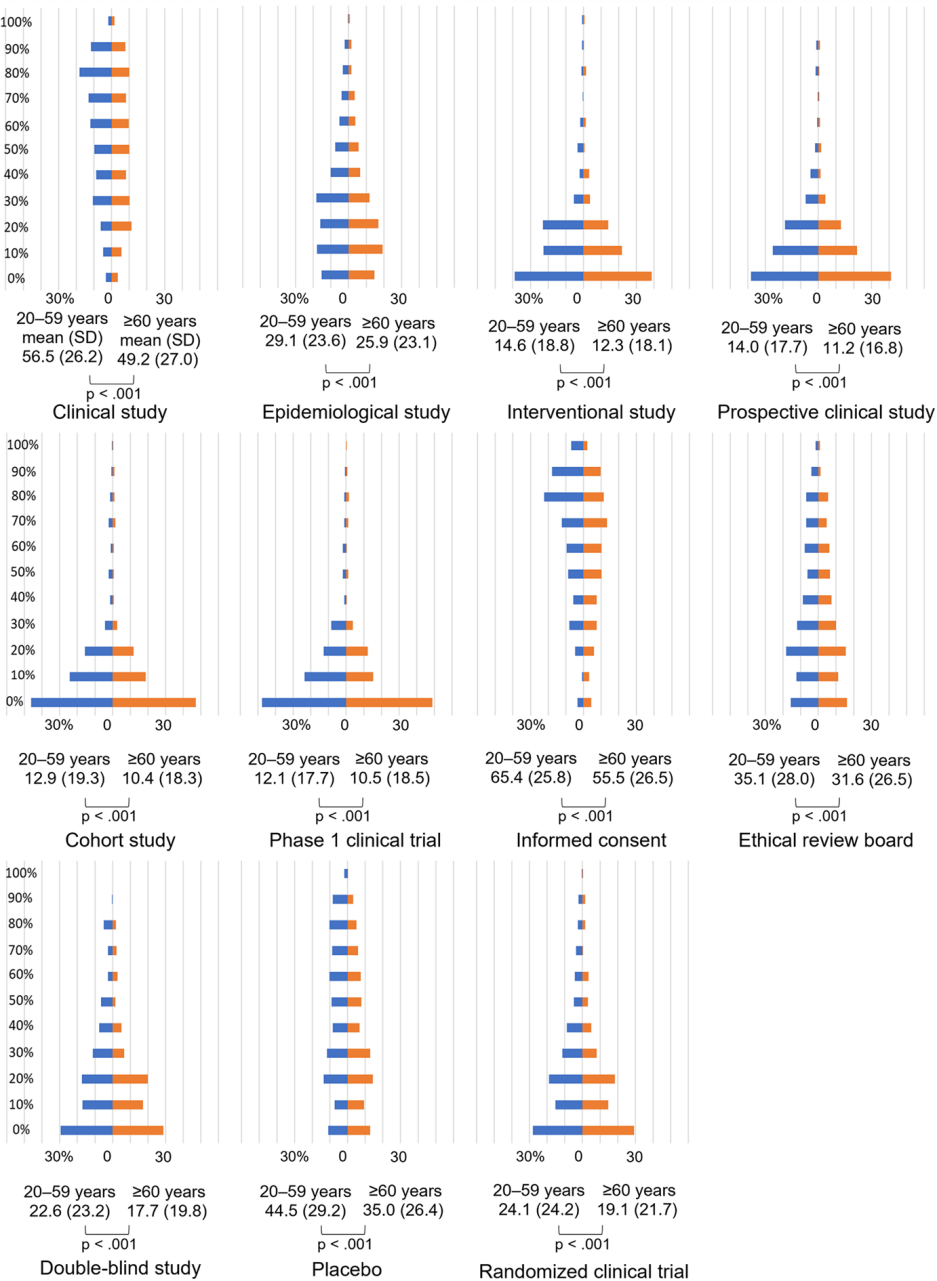
Physicians’ survey: Distribution of physicians’ prediction of public awareness by age group (*n* = 275). Percentages on the vertical axis indicate the proportion of the general public that physicians predicted to be “*likely understood*” and “*likely heard*” for each term, and percentages on the horizontal axis indicate the proportion of respondents. For example, the graph of *clinical study* shows that most physicians predicted that around 80% of the general public aged 20–59 would fall under “*likely understood*” or “*likely heard*”

The ranking of physicians’ predictions for the public awareness of terms is the same up to 9th place in both assumed age groups as follows: (1) *informed consent*, (2) *clinical study*, (3) *placebo*, (4) *ethical review board*, (5) *epidemiological study*, (6) *randomized clinical trial*, (7) *double-blind study*, (8) *interventional study*, and (9) *prospective clinical study*. However, the mean value of predicted awareness was less than 50%, even in the third-ranked term. In a comparison across assumed age groups, we observed that for all terms, physicians predicted that a statistically significantly higher proportion of the public in *the group of people under 60* would be familiar with each term than those in *the group of people 60s–70s* (Fig. 2. Details are presented in Supplementary Table 2).

We further analyzed whether physicians’ predictions were related to their experience of being involved in medical research on human subjects. Using the Mann–Whitney *U* test, physicians without experience provided higher predictions for *interventional study* for individuals in *the group of people under 60* and *cohort study* and *Phase 1 clinical trial* for individuals in *the group of people 60s–70s*. However, no significant differences were observed when compared using a *t*-test.

### Perception Gaps between Public Awareness and Physicians’ Predictions

**Fig. 3:**
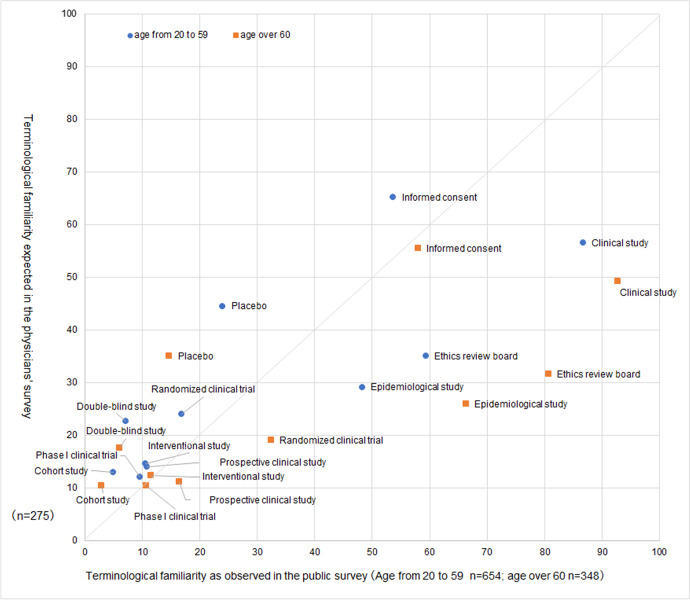
Scatterplot of terminological familiarity as observed in the public survey versus as estimated in the physicians’ survey. The region in the upper left of the graph (above the diagonal line) signifies terms whose expected familiarity in the physicians’ survey exceeded their actual familiarity in the public survey. On the contrary, the region to the lower right of the graph (below the diagonal line) specifies the opposite—terms whose expected familiarity in the physicians’ survey was underestimated compared to their actual familiarity in the public survey

To clarify the perception gaps between public awareness and physicians’ predictions, we plotted the results of the two surveys in Fig. 3. Each term is plotted separately for the public in the *group of people under 60* and *the group of people 60s–70s*. In the physicians’ survey, physicians estimated that, compared to *the group of people 60s–70s*, the public in *the group of people under 60* would be familiar with all surveyed medical research terms. However, the graph shows that this prediction applied to only a single term: *placebo*. Furthermore, on the one hand, we could know from the graph that the public less widely recognized four terms in comparison to the predictions by physicians (*placebo*, *cohort study*, *double-blind study* [both age groups], and *randomized clinical trial* [the *group of people under 60* only]). On the other hand, the four terms that generally matched the public awareness and the physicians’ prediction were *informed consent*, *prospective clinical study, interventional study,* and *Phase 1 clinical trial*. *Informed consent* was ranked third in the general public’s awareness in *the group of people under 60* and fourth in *the group of people 60s–70s*; however, it was ranked first in the physicians’ prediction regardless of the assumed age groups. In this way, there was a gap in the ranking, but there was no significant gap because the physicians’ prediction was low even if it was ranked in the first place.

## Discussion

From the public survey, we found that the only term that can be said to be popular among the general public in Japan is *clinical study*. Furthermore, the most gripping issue is that 80% or more of the respondents in *the group of people 60s–70s* indicated that they had never heard the following six terms: *interventional study*, *prospective clinical study*, *cohort study*, *Phase 1 clinical trial*, *double-blind study*, and *placebo*; and also *the group of people under 60* had not heard the following six terms: *interventional study*, *prospective clinical study*, *cohort study*, *Phase 1 clinical trial*, *double-blind study*, and *randomized clinical trial*. Several of these terms have been studied in previous research in Japan. For instance, *placebo* has been studied by both Yoshida et al. (2013) and Tsutani et al. (2015); the results of awareness obtained by these researchers (former: approximately 14.9%; latter: approximately 30% [males 30.6%, females 27.1%]) and our results (*the group of people under 60*: 24.0%; *the group of people 60s–70s*: 14.7% [overall 20.8%]) indicate roughly equivalent levels of awareness. Yoshida et al. also polled respondents about the terms *double-blind study* and *Phase 1 clinical trial*; 7.3% and 8.6% of respondents were aware of these terms, respectively, consistent with our results. In comparison to these findings, we can surmise that the results of this study are valid.

In this way, the general public’s awareness of the above six terms is low. If the physicians engaged in the research can predict this accurately, they will be conscious of the need to provide sufficient explanations of medical research terminology to the general public. Unfortunately, however, the physicians’ survey revealed that physicians could not make correct predictions. In the physicians’ survey, physicians estimated that, compared to *the group of people 60s–70s*, a greater percentage of the *group of people under 60* would be familiar with all surveyed medical research terms. However, most terms were more familiar to respondents in *the group of people 60s–70s* than those in the *group of people under 60*, indicating a gap between physician predictions and public awareness. This suggests the possibility that physicians are not providing the quality explanations that participants in *the group of people under 60* require. We assume one reason for the generally higher awareness among respondents in *the group of people 60s–70s* is as follows. In 2019, the average life expectancy in Japan was 81.41 years for men (third highest in the world) and 87.45 years for women (second highest in the world), and in general, Japanese people are very conscious of health and medical care. In a survey of men and women aged 55 years and older conducted by the Japanese government, 90.7% reported that they habitually engaged in health behaviors. One of these activities included “gaining knowledge about health and medical care,” which was endorsed by more than one-third (36.5%) of the respondents (Cabinet Office, Government of Japan 2017). Perhaps their attention is partially drawn to medical research due to this mindset. Physicians must reanalyze their perceptions.

There were four terms for which physicians predicted public awareness to be significantly higher than actual awareness: placebo, *cohort study*, *double-blind study* (both age groups), and *randomized clinical trial* (*the group of people under 60* only). These raise the possibility that the physician will not explain what participants need.

From these results, we found two factors that may hinder the proper IC in Japan: (1) many terms of medical research are not familiar to the general public, and (2) physicians generally fail to predict public awareness accurately. To remove these factors, it is considered that various measures should be taken, including the following.

First is “informing” the fact. It is critical to inform physicians about the general public’s low awareness of the six terms above and lesser familiarity (than assumed) with the following terms: *placebo*, *cohort study*, *double-blind study*, and *randomized clinical trial*. And also, informing those facts to research ethics committees (RECs) may be effective as they review informed consent forms. The second measure is “enhancing” the skill of physicians. Physicians should enhance their skills to explain medical research terminology to research subjects in an easy-to-understand manner. Many studies have been conducted to develop training programs to boost physicians’ communication skills for proper informed consent in medical research (Occa and Morgan 2018), and some of which have explored the explanation of the terms “randomization” and “placebo.” For example, Yap et al. recommended physicians for using metaphors like “flip of a coin,” “picking a number out of the hat,” “a roll of the dice,” and “luck of the draw” for “randomization” (Yap et al. 2009). However, as far as we know, no programs are focusing on the explanation of medical research terminology despite their need. Though the programs should be developed circumspectly, one idea would be to hold a workshop where physicians who are in charge of informed consent, the general public, and the patients (including ex-patients) who have experience participating in clinical research collaboratively discuss and create some samples of explanations text of terms. Through the workshop, it is expectable for physicians to learn whether the general public and patients can understand the explanations that are usually given, and what kind of information is needed for them. And then, the third measure is “sharing” the information. The created samples of explanatory text should be made public so that physicians and REC members can refer to them. In Japan, by relevant laws and guidelines, all persons involved in clinical research including physicians in charge of informed consent are obliged to undergo research ethics education programs before starting and during the research, and REC members are also required to take educational programs. In these circumstances, it is appropriate to incorporate those measures in such programs. Fortunately, we are conducting a project funded by a national research agency to produce video programs for researchers and REC members, respectively, and hold seminars on research ethics. We are willing to contribute to “informing” and “sharing” by producing video programs and “enhancing” by conducting seminars.

The last measure is “increasing” literacy. It is necessary to raise the general public’s awareness of medical research terminology and literacy of medical research. As mentioned above, six out of the eleven terms were unheard of by more than 80% of the general public. This means that most of the general public will come across these terms for the first time when they or their family members are ill and are briefed by a physician for a clinical study. Considering previous research, there are concerns that such a situation may cause two adverse effects on obtaining proper informed consent. One is that research subjects may feel fear of becoming “a guinea pig” (Behrendt et al. 2011; Quinn et al. 2007), similar to “a last chance for someone who has no hope” (Quinn et al. 2007) or a “death sentence” (Quinn et al. 2012). For instance, in a study by Naidoo et al. on patients that participated in a randomized clinical trial, when providing IC, respondents felt fear of becoming “guinea pigs,” intimidated and confused by specialized terminology, and disappointment, anger, and depression at the prospect of allocation into the control group of the study; in short, these processes were associated with psychological burdens (Naidoo et al. 2020). Further, unless a participant knows the facts, such as that many clinical studies are conducted for the development of medicine or that various methods such as randomized allocation are used in clinical studies to obtain reliable data, they may feel distrust toward their physicians. Another possible effect is that “therapeutic misconception” (Appelbaum et al. 1987; Henderson et al. 2006) is more likely to occur. In this way, even if the requirement (i), namely *information*, for proper IC is met, it may be difficult for the participant to fully understand the explanation and make a decision on participation in situations where they are proposed to participate in the medical research. In these circumstances, it is crucial to raise the general public’s literacy of medical research, including medical research terms. To consider the way to raise literacy, it is necessary to investigate why the Japanese public is unfamiliar with medical research terminology. In this regard, Japan ranks high in scientific literacy, having performed second-best out of 37 countries on the 2018 Program for International Student Assessment (PISA), a test conducted by the Organization of Economic Cooperation and Development (OECD) to evaluate learning attainment among 15-year-olds (OECD 2019). We speculate a major cause for this low familiarity with medical research terminology, despite high scientific literacy, is a lack of education. Japanese students are not taught the basic knowledge related to medical research in secondary education. For this, the national government should consider implementing education related to basic knowledge of medical research. Along with that, taking measures to raise public literacy should be taken immediately. For us, we will produce educational tools such as videos or leaflets using the explanation texts of the terms made in the second measure above so that young people can easily learn about medical research.

It is difficult to establish whether the medical research terminology remains largely unfamiliar to the general public in other countries, as a similar survey has not been conducted before. However, concerning the term *randomized clinical trial*, a survey carried out in the UK that targeted the general public found that only 28.6% of respondents had heard of the term (Mackenzie et al. 2010); this is similar to our result among Japanese people. Further, a 2020 public survey conducted by the American National Cancer Institute found that 36.4% of respondents indicated “I don’t know anything about clinical trials,” whereas 49.9% indicated “I know a little bit about clinical trials” (National Cancer Institute 2020). These findings suggest that even in countries where the clinical research is quite common and advanced, the general public’s awareness of clinical research is probably not high. Therefore, the measures above are worth considering not only in Japan but also in countries where the general public’s literacy in medical research is not high.

### Limitations

Our study has some limitations. First, our subjects were individuals registered with an online survey company. According to a 2018 investigation conducted by the Japanese government, the country’s Internet usage rate exceeded 90% in every adult age group between the 20s and 50s but declined in older demographics (60–64 years: 81.2%, 65–69 years: 67.9%, 70–79: 46.7%, ≥ 80: 20.1%) (Ministry of Internal Affairs and Communication 2018). For this reason, Japanese adults over 60 years of age who register with online survey companies have superior access to information than their peers in the same generation. Previous studies in the USA have shown that those registered with online survey companies have a relatively high educational background (Chang and Krosnick 2009). Though we could not analyze this factor since we did not collect information regarding the respondents’ educational backgrounds, there may be a similar tendency in Japan. In addition, it should also be noted that the response rate of the general public survey was as low as 12.8%. Online survey company registrants were requested to respond to various surveys, so that those who responded to our survey might have already been relatively interested in medical research. And also, we defined the “general public” as those who were not likely to touch with medical research terminology for occupational reasons, then excluded the candidate who worked or had family members working in several professions. Thus, some bias may be present in our sampling methodology for the so-called general public. Further, some bias is also inherent in our physician sample, as only physicians registered with the online survey company were enrolled. Moreover, the number of respondents under the age of 40 was significantly smaller, and that of respondents in their 50s and above was significantly higher than the actual age distribution of Japanese physicians. Those limitations indicate vulnerabilities regarding the representativeness of the population of responses. Hence, in the future, it is necessary to verify and reinforce the findings obtained from these surveys, for example, through qualitative research.

## Conclusions

This study surveyed familiarity with 11 basic medical research terms among the Japanese public and physicians’ predictions of familiarity. Our findings revealed that two factors might hinder the proper IC in Japan: (1) many terms of medical research are not familiar to the general public, and (2) physicians generally fail to predict public awareness accurately. To remove these factors, it is necessary for us to address the following four measures: “informing” the fact, “enhancing” the physicians’ explanation skill, “sharing” the information, and “increasing” the general public’s literacy on medical research.

### Supplementary Information

Below is the link to the electronic supplementary material.

Supplementary file1 (DOCX 20 kb)

Supplementary file2 (XLSX 15 kb)

## Data Availability

The datasets analyzed in the current study are available from the corresponding author on reasonable request.
