# Supplementary material for: Public Awareness of Medical Research Terminology in Japan, and the Accuracy of Physicians’ Predictions regarding that Awareness
Source: Asian Bioeth Rev. 2023 Mar 30;15(4):397–416. doi: 10.1007/s41649-023-00247-4 (PMC10555973; doi:10.1007/s41649-023-00247-4)
Supplement: Supplementary file 1 — Supplementary file1 (DOCX 20 kb) [file 41649_2023_247_MOESM1_ESM.docx]

**Supplementary Table1. Result details of public awareness by age group (*N*=1,002).**

|  | | Total | | Under 60 years (20-50 years) | | 60 years and above (≥60 years) | | *P-*value | odds ratio [95% [confidence](https://eow.alc.co.jp/search?q=confidence&ref=awlj) [interval](https://eow.alc.co.jp/search?q=interval&ref=awlj)] | Power |
| --- | --- | --- | --- | --- | --- | --- | --- | --- | --- | --- |
|  |  | n=1002 | | n=654 | | n=348 | |  |  |  |
|  |  | n | ％ | n | ％ | n | ％ |  |  |  |
| Clinical study | Understand / have heard | 890 | 88.8 | 567 | 86.7 | 323 | 92.8 | **.003** | 0.50  [0.31 - 0.82] | 0.85 |
|  | Never heard | 112 | 11.2 | 87 | 13.3 | 25 | 7.2 |  |  |  |
| Epidemiological study | Understand / have heard | 547 | 54.6 | 316 | 48.3% | 231 | 66.4% | **<.001** | 0.47  [0.36 - 0.62] | 1.00 |
|  | Never heard | 455 | 45.4 | 338 | 51.7 | 117 | 33.6 |  |  |  |
| Interventional study | Understand / have heard | 109 | 10.9 | 69 | 10.6 | 40 | 11.5 | .670 | 0.91  [0.59 - 1.39] | 0.06 |
|  | Never heard | 893 | 89.1 | 585 | 89.4 | 308 | 88.5 |  |  |  |
| Prospective clinical study | Understand / have heard | 128 | 12.8 | 71 | 10.9 | 57 | 16.4 | **.017** | 0.62  [0.42 - 0.91] | 0.75 |
|  | Never heard | 874 | 87.2 | 583 | 89.1 | 291 | 83.6 |  |  |  |
| Cohort study | Understand / have heard | 42 | 4.2 | 32 | 4.9 | 10 | 2.9 | .139 | 1.74  [0.82 - 3.82] | 0.39 |
|  | Never heard | 960 | 95.8 | 622 | 95.1 | 338 | 97.1 |  |  |  |
| Phase 1 clinical trial | Understand / have heard | 100 | 10.0 | 63 | 9.6 | 37 | 10.6 | .658 | 0.90  [0.58 - 1.38] | 0.11 |
|  | Never heard | 902 | 90.0 | 591 | 90.4 | 311 | 89.4 |  |  |  |
| Informed consent | Understand / have heard | 553 | 55.2 | 351 | 53.7 | 202 | 58.0 | .205 | 0.84  [0.64 - 1.09] | 0.34 |
|  | Never heard | 449 | 44.8 | 303 | 46.3 | 146 | 42.0% |  |  |  |
| Ethical review board | Understand / have heard | 669 | 66.8 | 388 | 59.3 | 281 | 80.7 | **<.001** | 0.35  [0.25 - 0.48] | 1.00 |
|  | Never heard | 333 | 33.2 | 266 | 40.7 | 67 | 19.3 |  |  |  |
| Double-blind study | Understand / have heard | 68 | 6.8 | 47 | 7.2 | 21 | 6.0 | .513 | 1.21  [0.70 - 2.06] | 0.14 |
|  | Never heard | 934 | 93.2 | 607 | 92.8 | 327 | 94.0 |  |  |  |
| Placebo | Understand / have heard | 208 | 20.8 | 157 | 24.0 | 51 | 14.7 | **<.001** | 1.84  [1.29 – 2.62] | 0.97 |
|  | Never heard | 794 | 79.2 | 497 | 76.0 | 297 | 85.3 |  |  |  |
| Randomized clinical trial | Understand / have heard | 223 | 22.6 | 110 | 16.8 | 113 | 32.5 | **<.001** | 0.42  [0.31 - 0.57] | 1.00 |
|  | Never heard | 779 | 77.4 | 544 | 83.2 | 235 | 67.5 |  |  |  |

*Fisher’s exact test was used for statistical comparisons. Significant results (*p*<0.05) are indicated in bold.

**We conducted post hoc power analysis with GPower 3.1. https://www.psychologie.hhu.de/arbeitsgruppen/allgemeine-psychologie-und-arbeitspsychologie/gpower
